# Reconstruction of an emergency thoracotomy wound with free rectus abdominis flap: Anatomic and radiologic basis for the surgical technique

**DOI:** 10.1186/1749-7922-5-12

**Published:** 2010-05-07

**Authors:** Kaitlyn Gilman, Kyros Ipaktchi, Ernest E Moore, Carlton Barnett, Raffi Gurunluoglu

**Affiliations:** 1 Department of Plastic and Reconstructive Surgery, Denver Health Medical Center, University of Colorado Health Sciences Center, 777 Bannock Street, Denver, 80204 Colorado USA; 2 Department of Orthopaedic Trauma, Denver Health Medical Center, University of Colorado Health Sciences Center, 777 Bannock Street, Denver, 80204 Colorado USA; 3 Department of Surgery, Denver Health Medical Center, University of Colorado Health Sciences Center, 777 Bannock Street, Denver, 80204 Colorado USA

## Abstract

An alcoholic 50-year-old male patient with a history of schizophrenia sustained stab wounds into both ventricles and left lung, and survived following an emergency department thoracotomy. The EDT wound, however became infected requiring serial debridements of soft tissue, rib cartilage and sternum. Regional flap options such as pectoralis major and latissimus dorsi muscle flaps could not be employed due to inadequate reach of these flaps. Additionally, bilateral transection of the internal mammary arteries during emergency thoracotomy eliminated the use of rectus abdominis muscles as pedicled flaps based on the superior epigastric vasculature. Therefore, the EDT wound was reconstructed by using the right rectus abdominis muscle as a free flap. The deep inferior epigastric vessels of the flap were anastomosed to the right internal mammary vessels proximal to their transection level in the third-forth intercostal space. The flap healed with no further wound complications.

## Background

The use of the emergency department thoracotomy (EDT) is invaluable in salvaging critically injured patients [1]. Patients with penetrating cardiac wounds associated with cardiac tamponade have the highest EDT success, while the overall survival rate of EDT is 7.4% [1].

The postoperative infection rate of EDT is not reported in the literature and we have no previous event at Denver Health Medical Center over the past 33 years. We present a 50- year-old male patient with an infected chest wall wound following an emergent anterolateral thoracotomy. Preoperative planning and management of this rare wound complication is reviewed in this report.

## Case Presentation

**Figure 1:**
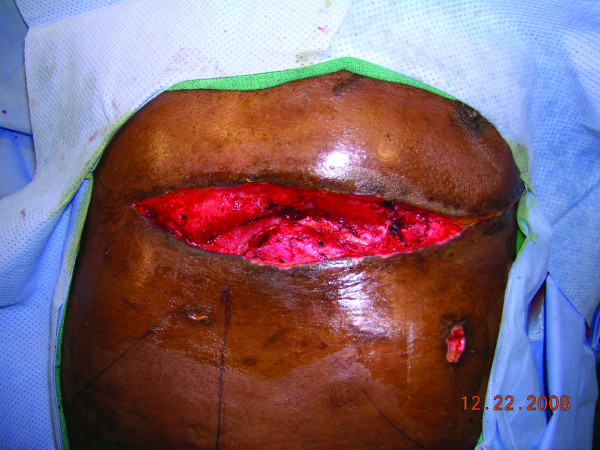
**Thoracotomy wound**: The thoracotomy wound after a serial debridement of soft tissue, rib cartilage and bone, and the sternum.

**Figure 2:**
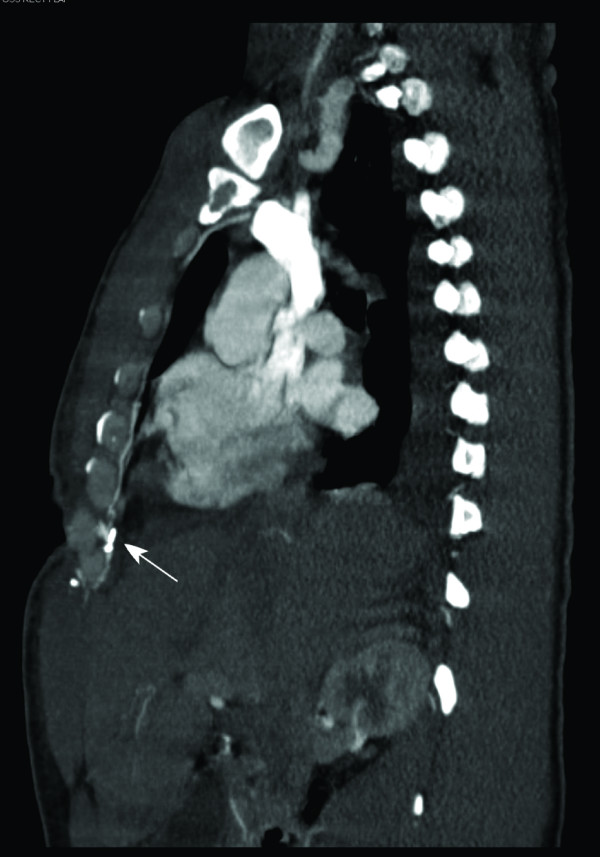
**Right sagittal CT angiography**: CT angiography (right sagittal section) performed for preoperative planning revealed interruption of the continuity of the right internal mammary vessels proximal to the surgical clip (arrow) at the level of the right seventh rib.

**Figure 3:**
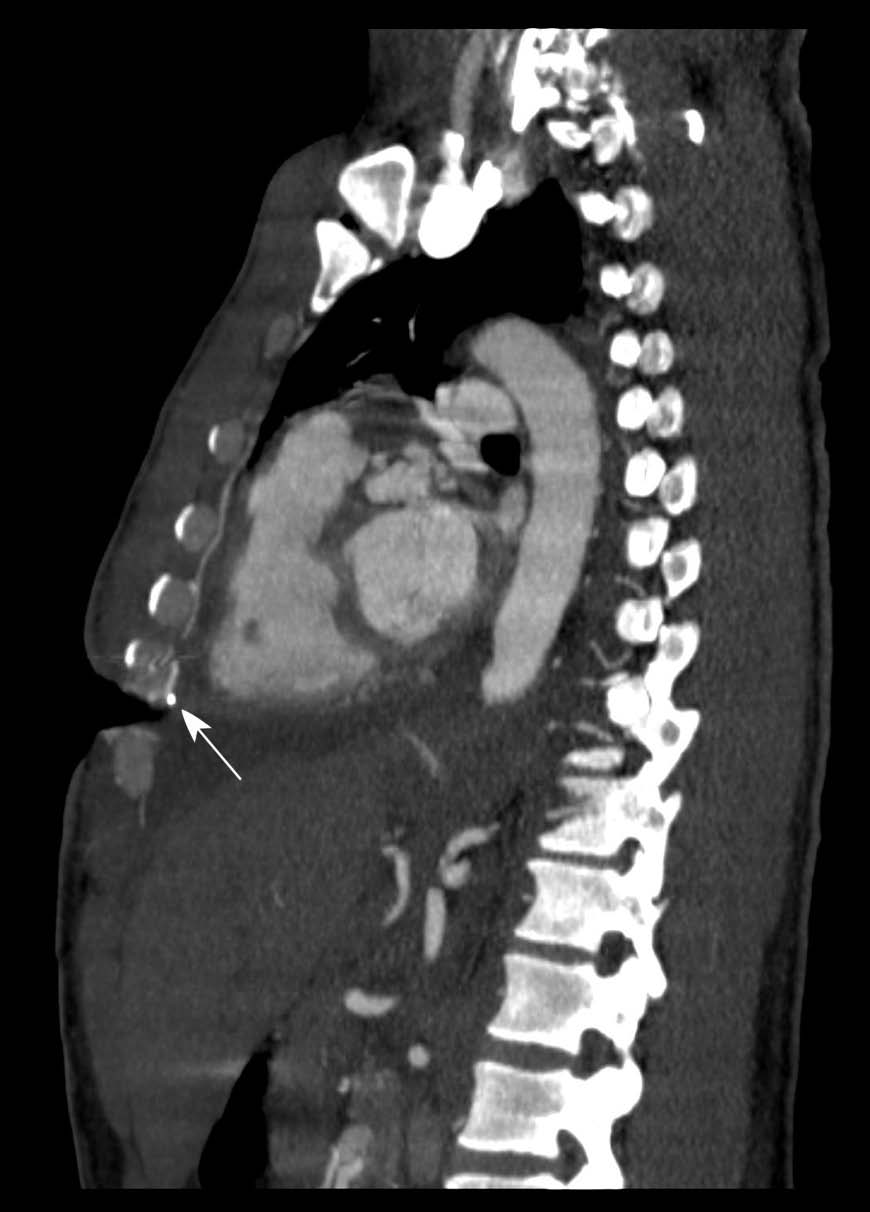
**Left sagittal CT angiography**: Preoperative CT angiography, left sagittal section also showed interruption of the continuity of the left internal mammary vessels proximal to the surgical clip (arrow) at the level of left fifth-seventh rib.

**Figure 4:**
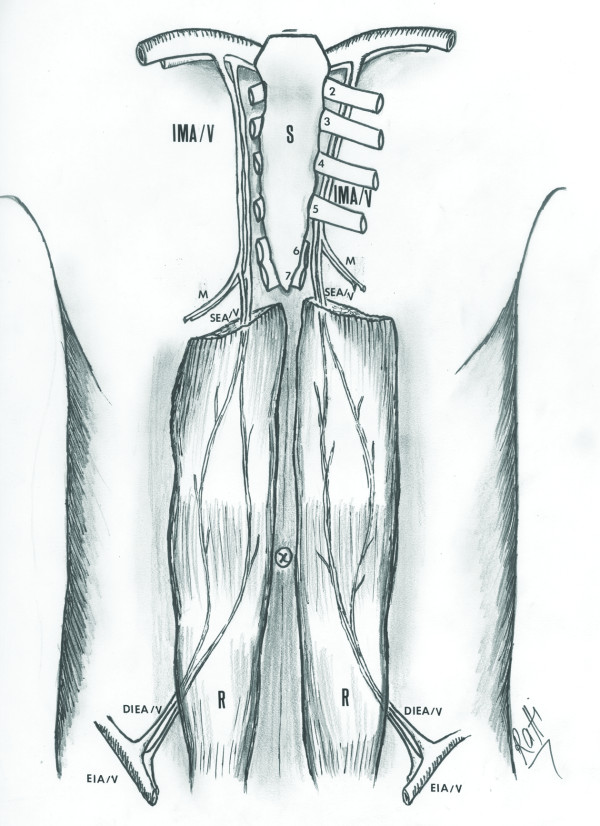
**The anatomical illustration of the rectus abdominis muscles, the superior epigastric artery, the internal mammary artery, and the deep inferior epigastric artery**: Line drawing that illustrates the anatomy of the rectus abdominis muscles, the superior epigastric artery, its relation with the internal mammary artery, and the deep inferior epigastric artery. The superior epigastric artery originates from the internal mammary artery at the level of the sixth and seventh rib. It then descends to enter the rectus sheath, at first behind the rectus abdominis muscle and then anastomoses with the deep inferior epigastric branch of the external iliac. IMA/V: The internal mammary artery and vein, SEA/V: The superior epigastric artery and vein, M: The musculophrenic branch, DIEA/V: The deep inferior epigastric artery and vein, EIA/V: The external iliac artery and vein, R: The rectus abdominis muscle, S: The sternum. Note that on the right side, the ribs have not been drawn to illustrate the course of the internal mammary vessels and their branching into the musculophrenic and the superior epigastric artery and vein. Additionally, the most proximal parts of the rectus abdominis muscles and first ribs on both sides have not been illustrated.

**Figure 5:**
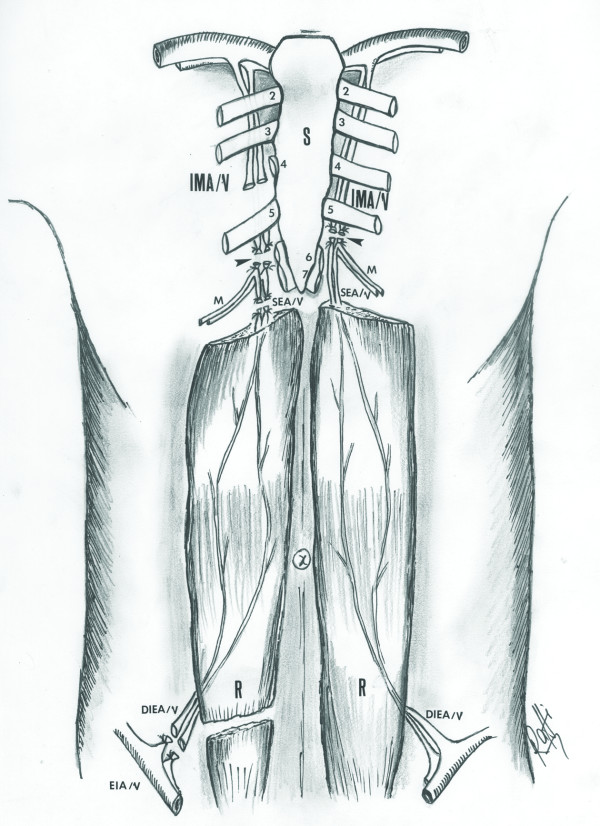
**The anatomical illustration of the IMA/V, the DIEA/V and SEA/V in the actual patient**: Line drawing to illustrate the anatomy of the IMA/V, the DIEA/V and SEA/V in the actual patient who underwent emergency thoracotomy with bilateral transection of the internal mammary vessels (arrow heads) prior to branching into the musculophrenic and the superior epigastric branches. Removal of the forth rib and preparation of the right IMA/V, DIEA/V and ligation of the right SEA/V for harvest of the rectus abdominis muscle for free transfer have been illustrated. IMA/V: The internal mammary artery and vein, SEA/V: The superior epigastric artery and vein, M: The musculophrenic branch, DIEA/V: The deep inferior epigastric artery and vein, EIA/V: The external iliac artery and vein, R: The rectus abdominis muscle, S: The sternum.

**Figure 6:**
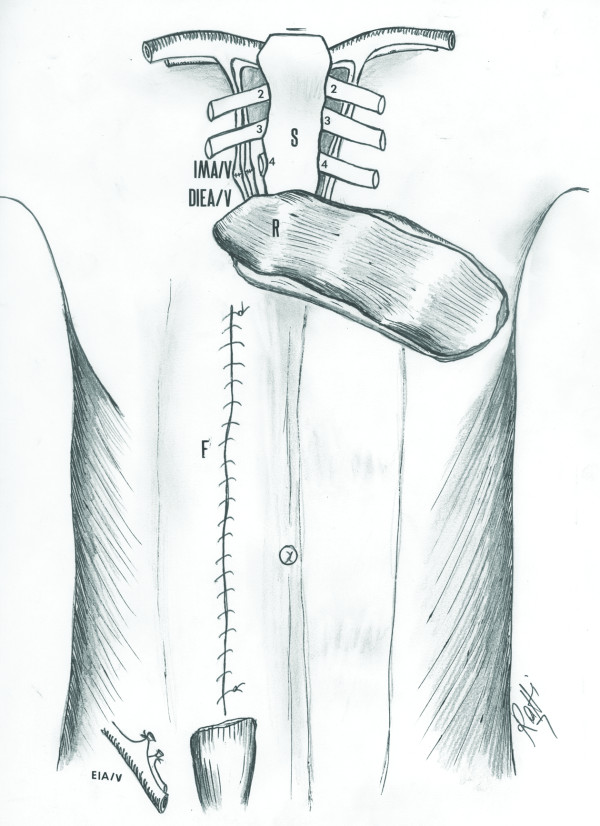
**Reconstruction of the wound with the free rectus abdominis muscle flap**: Line drawing illustrating the free rectus abdominis muscle transfer for thoracotomy wound reconstruction: The right internal mammary artery and vein were anastomosed in an end to end fashion to the right deep inferior epigastric artery and vein, respectively. IMA/V: The internal mammary artery and vein, DIEA/V: The deep inferior epigastric artery and vein, EIA/V: The external iliac artery and vein, R: The rectus abdominis muscle, S: The sternum, F: Fascial closure.

**Figure 7:**
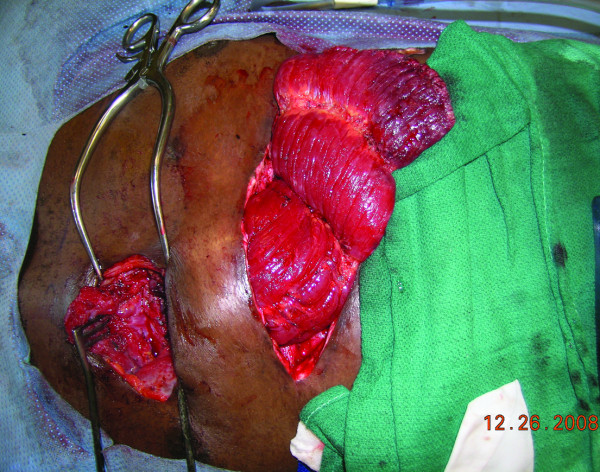
**The free rectus muscle transferred to the wound**: The free rectus abdominis muscle flap transferred to the wound. The right internal mammary vessels extending from the third to fourth intercostal space were prepared for microvascular anastomoses after removal of the third cartilaginous rib.

**Figure 8:**
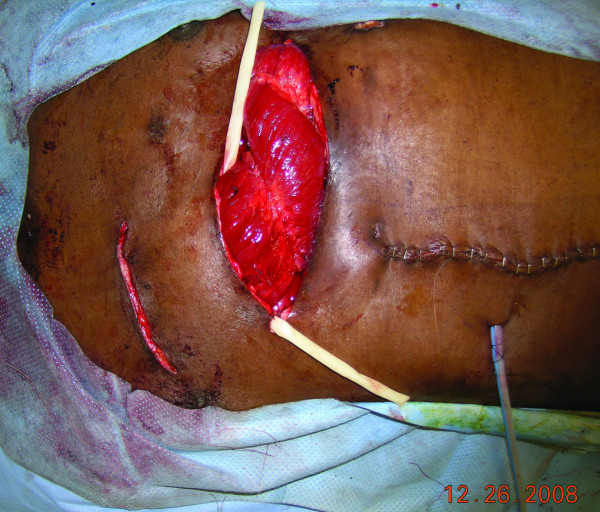
**The inset of the rectus muscle**: The right chest incision in the recipient site was closed and the free rectus muscle flap was inset.

**Figure 9:**
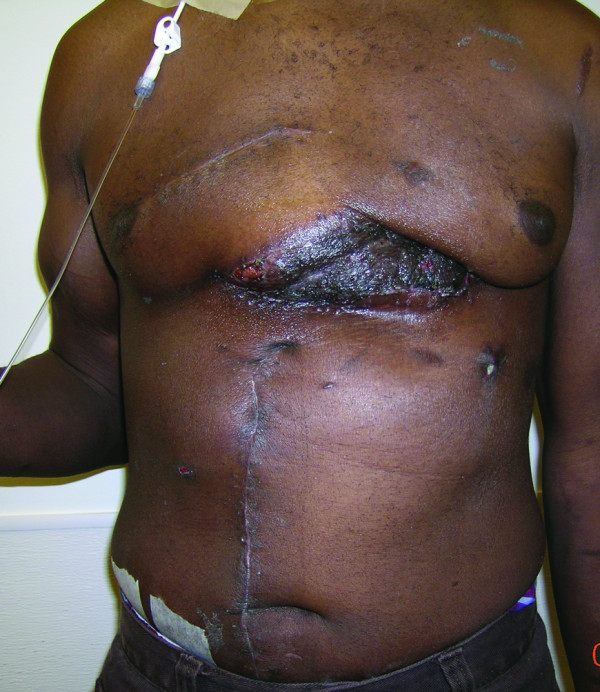
**Postoperative picture**: Two months after the reconstruction.

A 50-year-old alcoholic male with a history of schizophrenia presented in profound shock to the Denver Health Emergency Department with stab wounds to the left thorax. 1.5 liter of blood was aspirated with an emergent pericardiocentesis and the patient underwent resuscitative anterolateral thoracotomy in the ED. The emergency thoracotomy was performed in the standard fashion, with an incision made along the left fifth intercostal space extending across the sternum. After cardiac repair and hemostasis, the incision was closed primarily. At ten days post-operatively, the patient developed a thoracotomy wound infection that cultured positive for methicillin resistant staphylococcus aureus. Despite appropriate antibiotics, the infection necessitated radical debridement of involved bone (lower part of the sternum and rib), cartilage and soft tissue. Vacuum-assisted closure device (KCI, USA, San Antonio, TX) was placed after each debridement. The wound after two debridements measured approximately 20 × 8 cm, and extended deep to the pericardium (Figure 1). Location of the EDT wound however precluded use of pectoralis major or latissimus dorsi muscle flaps due to the inadequate reach of these flaps. A CT angiography of the internal mammary vasculature was performed to explore the potential use of a superiorly based rectus abdominis muscle flap for the wound reconstruction. However, it revealed interruption of the contrast medium in the internal mammary vasculature at the level of the right seventh rib (Figure 2) and left fifth-seventh rib (Figure 3). Therefore, a free tissue transfer by using the right-sided rectus abdominis muscle flap was carried out for wound reconstruction. The deep inferior epigastric vessels, the vascular pedicle of the flap, were anastomosed to the right internal mammary vessels in the third-forth intercostal space proximal to the transection level for restoration of perfusion [2] (Figure 4, 5, and 6). The surface of the muscle flap was skin grafted. The flap took successfully and the patient healed without further complications (Figures 7, 8, and 9).

## Discussion

Wound complications associated with emergency thoracotomy have not been reported in the literature. In light of the almost non-existent infection rate, surgical debridement and the reconstruction of EDT wounds is rarely necessitated. The management of the complicated EDT wound was initiated by adequate surgical debridement and appropriate antibiotic treatment prior to definitive reconstruction. In addition, coverage especially with a muscle flap was planned to overcome the infection and to supplement the healing in such a wound with exposed heart.

The pectoralis major, the latissimus dorsi, the rectus abdominis, and omental flap are most frequently employed flaps in the chest and sternal region wound reconstruction [3,4]. However, in our case, reconstruction of the thoracotomy wound presented several reconstructive challenges. The pectoralis major or latissimus dorsi muscle flaps were not suitable with regards to the location of the EDT wound. The omental flap was not employed to avoid laparotomy and associated risks. On the other hand, the rectus abdominis muscle could not be utilized since the superior epigastric vessels, the pedicle of a superiorly based flap, were found to be unreliable.

The superior epigastric artery originates from the internal mammary artery at the level of the seventh rib. Then, it descends between the costal and xiphoid slips of the diaphragm, anterior to the lower fibers of the transversus thoracis and transversus abdominis. Entering the rectus sheath, at first behind the rectus abdominis muscle and then perforating and supplying it, it anastomoses with the deep inferior epigastric branch of the external iliac [5] (Figure 4).

The EDT procedure involved a knife incision along the inferior border of the pectoralis major muscle at the level of fourth or fifth intercostal space running from the border of the sternum to mid-axillary line. In our case, extension across the sternum to the right hemithorax was required for exposure of pleural, anterior, and mediastinal structures. Horizonal transection of the sternum during EDT required ligation of the internal mammary arteries, which lie approximately 1.57 ± 0.30 cm lateral from the right and 1.47 ± 0.30 cm lateral from the left of the sternal edge [6].

Bilateral transection of the internal mammary vessels proximal to the terminal bifurcation during an EDT interrupted the superiorly based blood supply of the both rectus abdominis muscles, precluding the possibility of a superiorly based rectus abdominis flap from either side for wound reconstruction (Figure 5). Therefore, we addressed the given limitations by utilizing a free flap reconstruction of the EDT wound. Because of the suitability with regards to its dimensions, proximity to the defect, and large caliber vascular pedicle, the rectus abdominis muscle was used as a free flap for wound reconstruction. The right internal mammary vessels proximal to the transection level were anastomosed to the deep inferior epigastric vessels (dominant pedicle) of the flap for perfusion.

In the event of rare EDT wound complication requiring reconstruction, the integrity and patency of the internal mammary vasculature must be carefully assessed for the potential use of rectus abdominis muscles as a pedicled flap. Nevertheless, the possibility of using the rectus abdominis flap based on the superior epigastric vasculature would be remote in most cases, other flaps such as pectoralis major and latissimus dorsi flaps will not reach to the wound and reconstructive surgery by using free tissue transfer would be required.

## Competing interests

The authors declare that they have no competing interests.

## Authors' contributions

**KG**: has been involved in drafting the manuscript.

**KI**: assisted the free flap reconstruction surgery.

**EEM**: revised the manuscript critically for important intellectual content.

**CCB**: performed the emergency thoracotomy.

**RG**: performed the free flap reconstruction surgery, contributed significantly to design of the case report and gave final approval of the version to be published.

All authors read and approved the final manuscript.

Written informed consent was obtained from the patient for publication of this case report and accompanying images. A copy of the written consent is available for review by the Editor-in-Chief of this journal.

## References

[B1] Cothren CC Moore EE Emergency department thoracotomy for the critically injured patient: objectives, indications, and outcomes World J Emerg Surg 2006 1 4 10.1186/1749-7922-1-4 16759407 PMC1459269

[B2] Ninkovic MM Schwabegger AH Anderl H Internal mammary vessels as a recipient site Clin Plast Surg 1998 25 213 221 9627779

[B3] Davison SP Clemens MW Armstrong D Newton ED Swartz W Sternotomy wounds: Rectus flap versus modified pectoral reconstruction Plast Reconstr Surg 2007 120 929 34 10.1097/01.prs.0000253443.09780.0f 17805121

[B4] Roth DA Aston, SJ, Beasley RW, Thorne CHM Thoracic and abdominal wall reconstruction Grabb and Smith's Plastic Surgery 1997 Philadelphia: Lippincott-Raven Publishers 1023 1029

[B5] Williams PL Warwick R Dyson M Bannister LH eds Angiology Gray's Anatomy 1989 37 New York: Churchill livingstone 754 755

[B6] Glassberg RM Sussman SK Glickstein MF CT anatomy of the internal mammary vessels: importance in planning percutaneous transthoracic procedures AJR Am J Roentgenol 1990 155 397 400 2115273 10.2214/ajr.155.2.2115273

